# Overexpression of lncRNA TUG1 Alleviates NLRP3 Inflammasome-Mediated Cardiomyocyte Pyroptosis Through Targeting the miR-186-5p/XIAP Axis in Coronary Microembolization-Induced Myocardial Damage

**DOI:** 10.3389/fimmu.2021.637598

**Published:** 2021-06-07

**Authors:** You Zhou, Tao Li, Zhiqing Chen, Junwen Huang, Zhenbai Qin, Lang Li

**Affiliations:** ^1^Department of Cardiology, The First Affiliated Hospital of Guangxi Medical University, Guangxi, China; ^2^Guangxi Key Laboratory of Precision Medicine in Cardio-cerebrovascular Diseases Control and Prevention, Guangxi, China

**Keywords:** coronary microembolization, NLRP3 inflammasome, pyroptosis, lncRNA TUG1, miR-186-5p, XIAP

## Abstract

Coronary microembolization (CME) is a complicated problem that commonly arises in the context of coronary angioplasty. The lncRNA taurine-up regulated gene 1 (TUG1), significantly contributes to cardiovascular diseases; however, its contribution to CME-induced myocardial damage remains elusive. Herein, we establish the rat CME model and investigate the role of TUG1 in CME. The cell viability was evaluated via CCK-8 assay. Serum and cell culture supernatant samples were evaluated via ELISA. The dual luciferase reporter (DLR) assay, RIP, and RNA-pull down were conducted to validate the associations between TUG1 and miR-186-5p as well as miR-186-5p and XIAP. The expression of TUG1, miR-186-5p, and XIAP mRNA were determined by RT-qPCR, and proteins were evaluated via immuneblotting. As a result, TUG1 and XIAP were significantly down-regulated, and the miR-186-5p level was found to be remarkably up-regulated in CME myocardial tissues. Overexpression of TUG1 alleviated CME-induced myocardial injury and pyroptosis, whereas TUG1 knockdown showed the opposite effects. The DLR assay, RIP, and RNA-pull down results reveal that TUG1 directly targets miR-186-5p and miR-186-5p directly targets XIAP. In vitro rescue experiments show that TUG1 overexpression alleviates LPS-caused cardiomyocyte injury and pyroptosis via sponging miR-186-5p and regulating XIAP, and depression of miR-186-5p reduces LPS-induced cardiomyocyte injury and pyroptosis by targeting XIAP. Concludingly, the overexpression of TUG1 alleviates NLRP3 inflammasome-mediated cardiomyocyte pyroptosis through targeting the miR-186-5p/XIAP axis in CME-induced myocardial injury.

## Introduction

Coronary microembolization (CME) is the most predominant complication in percutaneous coronary intervention (PCI) therapy (1, 2). The risk of CME in perioperative PCI has been revealed as up to 45% in high-risk patients (3). CME may result in myocardial malfunction, including myocardial infarction, arrhythmias, heart attack, and reduction of coronary flow reserve fraction (4–6). Numerous studies report the myocardial damage caused by CME, but the underlying molecular mechanisms for CME are still unclear.

Pyroptosis is an inflammatory-mediated cell death, which is stimulated via downstream-activated caspase-1 and ultimately results in cellular rupture and the release of proinflammatory cytokines (7, 8). Pyroptotic cell death is tightly regulated and has been linked to the incidence of a range of cardiovascular diseases (CVDs) (9–11). Therefore, suppressing cardiomyocyte pyroptosis might be a candidate target for CME. However, the molecular mechanisms governing cardiomyocyte pyroptosis in the context of CME are not clearly understood.

Long non-coding RNAs (lncRNAs) are a series of RNAs comprising more than 200 nucleotides that are not able to encode proteins. Initially, lncRNAs were considered to be “garbage” generated during gene expression, but emerging evidence indicates that lncRNAs contribute to many processes of the cell, including genomic imprinting, evaluation of cell fate, alternative splicing of RNA, and chromatin modification (12, 13). Currently, many lncRNAs are reported as significantly contributing to the pathogenesis of CVDs (14–16). The lncRNA taurine-up regulated gene 1 (TUG1) was observed during genomic screening in taurine-exposed retinal cells that are much conserved in mammals (17). Current studies reveal the dynamic expression of TUG1 in the progression and diseases of the heart (18, 19). It is, however, not known whether TUG1 may also regulate CME-induced myocardial damage and, if so, what mechanisms are involved.

In the current study, we determined the role of TUG1 in CME-induced myocardial injury and investigated the basic mechanism underlying this process. This study might give deeper insight into regulatory mechanisms underlying CME and provide a candidate target for CME.

## Materials and Methods

### Animal Modeling and Grouping

The *in vivo* studies were based on suggested procedures of the U.S. National Institutes of Health (NIH Publication No. 85–23, revised 1996). The study was approved by the Animal Care and Use Committee of Guangxi Medical University, China. Male Sprague–Dawley rats having a body weight of about 300 grams and age of 3 months were used. Controlled humidity of 50–60% and temperature of 23°C ± 2°C were maintained. Food and water were provided to rats in a fully maintained environment of 12:12 h light and dark periods. The modeling process for the CME model can be described as follows. Pentobarbital hydrochloride of 30~40 mg/kg was provided by an intraperitoneal (IP) injection under anesthesia, followed by tracheotomy and intubation. A mini animal ventilator was employed to facilitate respiration. The chest was exposed between the 3rd and 4th ribs on the left side of the breastbone (sternum). After opening the chest, the ascending aorta was exposed and clamped for 10 s using a vascular clip. This was followed by the injection of a mixture of 100 μl normal saline and 8,000 polyester microspheres with 42 μm diameter (Biosphere Medical Inc., Rockland) into the left ventricle via the apex of the heart with a micro syringe. After sucking out the residual liquid in the chest cavity, the chest was closed layer by layer, followed by IP administration of penicillin (80,000 units). The rat was then placed on a pad set at a constant temperature of 36.5°C. The tracheal intubation was removed when the breathing of the rat was stable. Similar surgery was conducted via injecting 100 μl normal saline into the left ventricle of the Sham group animals. The blood and heart samples were collected from the Sham as well as the CME groups at various time intervals, i.e., 3, 6h, 9, and 12 h, and the samples from the remaining groups were harvested 9 h after CME modeling. Heart samples were taken from the left ventrical near the apex in all groups. The fixing of heart samples was carried out in paraformaldehyde (4%) or instantly kept in liquid nitrogen for further analysis.

### Adeno Associated Virus (AAV), antagomiR and agomiR Treatment in Rats

All AAVs, antagomiR, and agomiR were generated via Gene Chem (Shanghai, China). To assess the biological effects of TUG1 and X-linked inhibitor of apoptosis protein (XIAP), a total titer of 10^11^ TU AAV-pcDNA3.1-TUG1 vs. AAV- pcDNA3.1-NC, AAV-shTUG1 vs. AAV-SHNC, or AAV- pcDNA3.1-XIAP vs. AAV- pcDNA3.1-NC was diluted with 200 μL *in vivo* transfection reagents (Engreen Biosystem, Beijing, China). The mixture was then injected into the vein in the rat tail 3 weeks before modeling.

To examine the functional role of miR-186-5p in CME-induced myocardial injury, antagomiR (a 2′OME+5′chol-modified miR-186-5p inhibitor) or miR-186-5p agomiR (a 2′OME+5′chol-modified miR-186-5p agonist) was used to regulate the expression level of miR-186-5p in pigs. In brief, we first prepared a nucleic acid dilution by dissolving 100 μg of miR-186-5p antagomiR (agomiR) or control antagomiR (agomiR) in 100 μL of a sterilized double-distilled H_2_O. Then, we mixed the solution well. Next, the nucleic acid dilution and *in vivo* transfection reagent (Engreen Biosystem, Beijing, China) were mixed (1:1) to form a working solution. At seven days before modeling, 200 μL miR-186-5p antagomiR/agomiR transfection complex or NC antagomiR/agomiR working solution was infused into the tail vein.

### Cell Transfection With TUG1-Expressing Plasmids, miR-186-5p-agomiR, miR-186-5p-antamiR, and XIAP SHRNA

The TUG1-expressing plasmids (pcDNA3.1-TUG1 plasmids) and corresponding control plasmids (pcDNA3.1-NC plasmids), miR-186-5p-agomiR and corresponding control agomiR (ago-NC), miR-186-5p-antagomiR and corresponding control antagomiR (anta-NC), XIAP SHRNA (SHXIAP), and a control SHRNA (SHNC) were purchased from Gene Pharma (Shanghai, China) and transfected into H9C2 cardiomyocytes using Lipofec-tamine™ 3000 (Life Technologies, USA) following the manufacturer's instructions. The cells were collected after 72 h of interference.

### Transmission Electron Microscopy (TEM)

From each group, the myocardial tissues were sliced into small pieces (1 mm^3^), followed by fixing with glutaraldehyde (2.5%) at 4°C for 24 h. Next, cleaning and dehydration of samples were carried out, followed by fixing and staining. Then, the samples were observed under a microscope, i.e., TEM (Hitachi H-7650, Japan) at x 30,000 magnification.

### Measurement of Myocardial Microinfarct Size

The red-stained section (ischemia or necrotic) evaluation was carried out via a DMR-Q550 pathological picture analyzer (Lei, Germany). The five vision fields (×200 magnification) were randomly chosen from individual haematoxylin-basic fuchsin-picric acid (HBFP) staining sections for evaluation of the infarction area via plane procedure with Leica Qwin analysis software.

### Detection of Cardiac Function (Philips Sonos7,500 Series, USA)

The LVEF parameter was calculated while keeping the probe frequency at 12 MHZ. The obtained values were the mean of three cardiac cycles. Echocardiographic results were calculated and evaluated via blinding to various therapies.

### Determination of c-troponin I (cTnI), LDH, IL-1β, and IL-18 Levels

The concentrations of CTNI LDH, IL-18, and IL-1β in serum samples or cell-free culture supernatant were assayed via ELISA kits (R&D Systems, USA) following the instructions of the manufacturer.

### Fluorescence *in situ* Hybridization (FISH)

The RNA scope 2.0 (ACD, Canada) assay was conducted for *in situ* evaluation of lncRNA TUG1 in the heart of the rat. The heart tissues were fixed with formalin (10%) for 0.5 h at 25°C, followed by protease digestion, and then hybridizations with probes were carried out that target TUG1. This was followed by counterstaining of tissues with DAPI, and the fluorescence signals were imagined via ZEISS LSM 880 Confocal Laser Scanning Microscope.

### RNA Isolation and QPCR Analysis

The total RNA was extracted from the LV myocardium via TRIzol following the instructions of the manufacturer. The RNA concentration was evaluated via Nano Drop. CDNA was synthesized with the help of a reverse transcription kit (TaKaRa, Japan). The obtained products of PCR were evaluated via SYBR-Green I. The relative gene expression was identified via the 2^−Δ*ΔCT*^ formula. The gene-specific primers were as follows: TUG1 primers: upstream: 5′- TTA AGG GCC AAA CGC CAT CA-3′, downstream: 5′- GGG CCA GTT GGG TAT AGC AG-3′; miR-186-5p primers: upstream: 5′- ACA CTC CAG CTG GGC AAA GAA TTC TCC TTT-3′, downstream: 5′- CTC AAG TGT CGT GGA GTC GGC AA-3′; XIAP primers: upstream: 5′- TCG GGC TGC ATA ATG AGG ACT G-3′, downstream: 5′- CCT TTT CGC GCC AAG CAA TC-3′; NLRP3: upstream: 5′- CTC TGC ATG CCG TAT CTG GT-3′, downstream: 5′- CAC CTC TTG CGA GGG TCT TT-3′; IL-1β primers: upstream: 5′- GAC TTC ACC ATG GAA CCC GT-3′, downstream: 5′- GGA GAC TGC CCA TTC TCG AC-3′; IL-18 primers: upstream: 5′- CAA CCG CAG TAA TAC GGA GC-3′, downstream: 5′- TCT GGT CTG GGA TTC GTT GG-3′; U6 primers: upstream: 5′- GCT TCG GCA GCA CAT ATA CTA AAA T-3′, downstream: 5′- CGC TTC ACG AAT TTG CGT GTC AT-3′ and GAPDH primers: upstream: 5′- GGA AAC CCA TCA CCA TCT TC-3′, downstream: 5′- GTG GTT CAC ACC CAT CAC AA-3′.

### Immunoblotting

Total myocardial protein from the apex of the left ventricle was extracted using protein lysis buffer (Beyotime Biotechnology, Beijing, China), and the Lowry method was used to determine the protein concentration. Equal amounts of protein (50 μg) were extracted via 10–15% SDS-PAGE and transferred onto PVDF membranes (Millipore, USA). The skimmed milk (5%) was used for the blockage of the membrane for 60 min at 25°C. The membrane was then incubated with primary antibodies at 4°C for 24 h. The primary antibodies specific to XIAP, GSDMD, IL-1β, IL-18, and GAPDH were provided by Abcam (Cambridge, UK). The specific primary antibodies for NLRP3, caspase-1 p20, and ASC were procured from Bioss (Beijing, China), followed by washing three times with TBST. The membrane was then incubated with secondary antibodies fused with horseradish peroxidase in TBST for 60 min at 25°C. An enhanced chemiluminescence detection system (Santa Cruz, CA, USA) was considered for the identification of the signals. Finally, Image Lab (Bio-Rad, USA) software was used for the evaluation of protein bands.

### Bioinformatics Analysis and DLR Assay

The Star Base V2.0 and Target Scan software were employed to evaluate the potential binding sequences within TUG1 and the 3' UTR of XIAP to miR-186-5p. A Site-Directed Mutagenesis kit (Stratagene, La Jolla, CA, USA) was used for the insertion of mutations into the candidate binding sequences. psiCHECK-2 vector (Promega, Madison, WI, USA) was used for cloning of the wild-type (WT) or mutant (Mut) binding sequences of TUG1 or XIAP and then co-transfected into primary cardiomyocytes with miR-186-5p mimic or MIR-NC (Gene Chem, Shanghai, China) via Lipofectamine 2,000, as suggested by the manufacturer, and after 2 days, DLR activity was evaluated.

### RNA-Binding Protein Immune Precipitation

The Ago2 antibody (Abcam, Cambridge, UK) and Magna RIP RNA-binding protein immune precipitation kit (Millipore, USA) were utilized to conduct RIP experiments. QRT-PCR was employed to evaluate the co-precipitated RNAs.

### RNA-Pull Down Assay

Cardiomyocyte transfection was performed with biotinylated miR-186-5p after 2 days, and then cells were harvested. The incubation of cell lysates was conducted with the help of M-280 streptavidin magnetic beads (Invitrogen, USA). To perform the QRT-PCR analysis, TRIzol reagent (Invitrogen, USA) was utilized for the purification of bounded RNAs.

### Cell Culture and Treatment

LPS and H9C2 cell lines were provided by ATCC, Rockville, USA. The H9C2 cell lines were seeded in DMEM provided with FBS (10%), streptomycin (100 μg per ml), and penicillin (100 U per ml). Next, the cell cultures were incubated in the presence of 5% CO_2_ supply at 37°C, followed by exposure of cardiomyocytes to LPS (10 μg/ml) for 12 h for the induction of inflammatory injury.

### Cell Viability Assay

The total number of live cells was analyzed via the CCK-8 kit (Dojindo, Japan). The CCK-8 kit was employed according to the provided guidelines of the kit supplier.

### Statistical Analysis

The results were displayed as means ± SEM. A Student *t*-test was considered to assess the variations between two independent groups. For multiple groups, statistical differences were evaluated via one-way ANOVA followed by the Bonferroni test. All the experiments were performed in triplicate. *P*-value < 0.05 was considered statistically significant. All calculations were made using SPSS 20.0.

## Results

### Lnc RNA TUG1 Expression Patterns in Myocardial Samples From CME Model Rats

**Figure 1:**
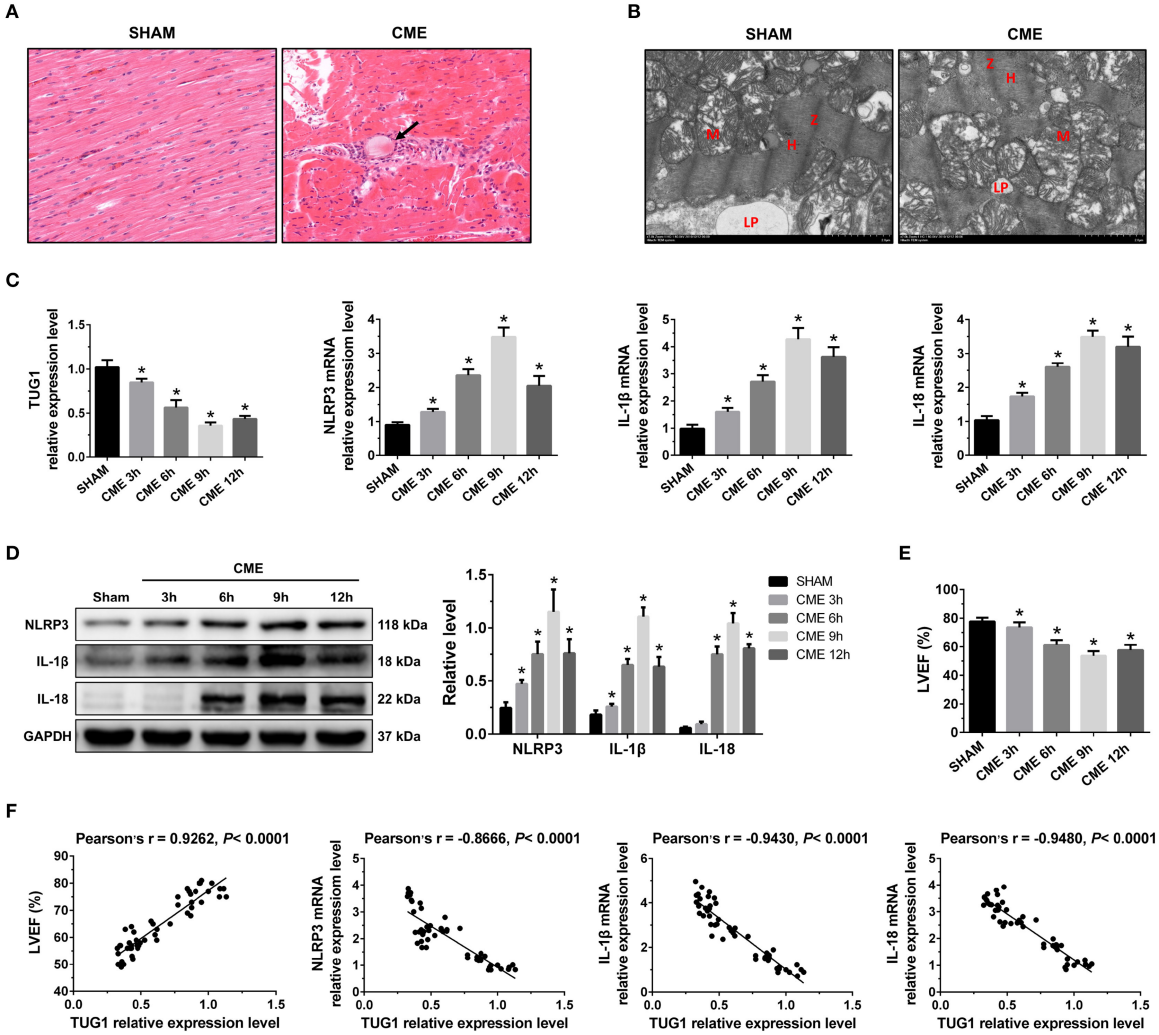
The expression pattern of lncRNA TUG1 in myocardial tissue following CME. **(A)** Myocardial tissue samples stained with hematoxylin and eosin (H&E). Microspheres are marked using black arrow (×200; scale bar = 50 μm). **(B)** Myocardial tissue samples were assessed via TEM (×30,000; scale bar = 2μm). M, mitochondria; LP, lipid droplets; H, H-bands; Z, Z line. **(C)** Myocardial TUG1, NLRP3, IL-1β, and IL-18 mRNA levels were assessed via qRT-PCR (*n* = 10). **(D)** Levels of myocardial NLRP3, IL-1β, and IL-18 protein were assessed via Western blotting. GAPDH served as a normalization control (*n* = 3). **(E)** Echocardiography was used to assess cardiac function and to quantify LVEF (*n* = 10). **(F)** TUG1 levels were positively correlated with LVEF and negatively correlated with NLRP3, IL-1β, and IL-18 mRNA expression. Data are mean ± SEM from three or more independent experiments. ^*^P < 0.05 vs. SHAM group.

A rat CME model was established using microspheres, which were visible in the myocardial tissue following injection. Inflammatory cell infiltration, disordered or ruptured myocardial fibers, and interstitial edema were visible surrounding the microspheres in CME model animals (Figure 1A). The analyses of TEM established that myocardial sarcomeres were partially ruptured and disordered following CME with unaligned Z-rays, mitochondrial vacuolation and s welling, and damaged mitochondrial cristae consistent with pyroptotic cell death (Figure 1B). Together, these findings confirm the successful establishment of a rat CME model.

In the CME group, the expression of TUG1 in myocardial tissue was considerably decreased compared with the Sham group, and the expression of NLRP3, IL-18, and IL-1β mRNA was significantly elevated (Figure 1C). The expression of these mRNAs reached the lowest or peak value at 9 h post-CME. In the CME group, the expression of NLRP3, IL-18, and IL-1β protein in the myocardium was also elevated significantly compared with the Sham group and reached the peak value at 9 h post-CME (Figure 1D). The CME group revealed a significant decline in LVEF, and the functions of the heart had the worst value at 9 h post-CME (Figure 1E). The analysis of Pearson's correlation demonstrated that TUG1 expression was considerably associated with LVEF, but negatively correlated with the expression of NLRP3, IL-18, and IL-1β mRNA (Figure 1F). These results reveal that TUG1 may contribute to CME-activated myocardial injury and pyroptosis. Thus, 9 h post-CME was selected as the time point for subsequent experiments.

### Lnc RNA TUG1 Overexpression Alleviates Myocardial Injury and Pyroptosis Following CME

**Figure 2:**
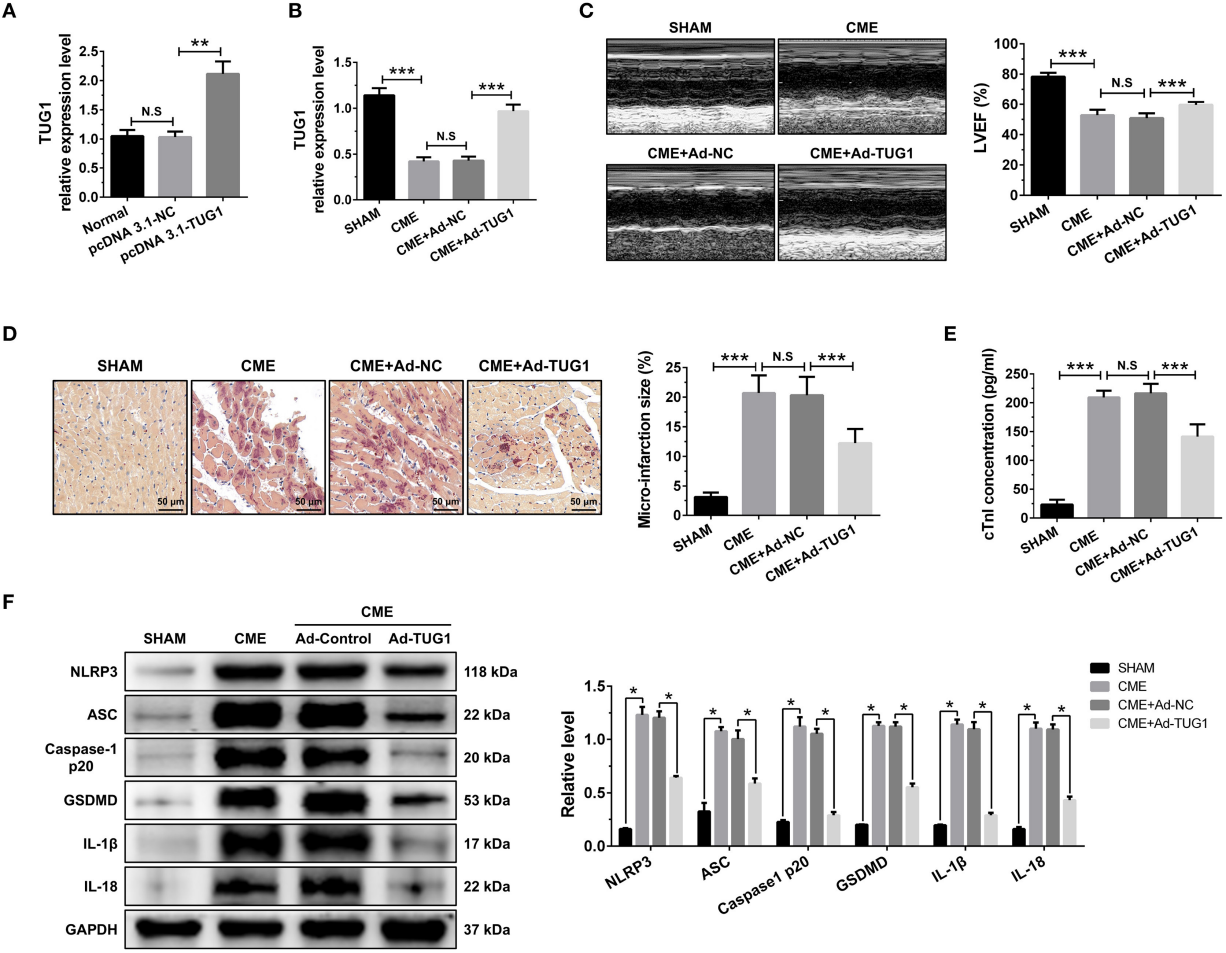
TUG1 overexpression reduces myocardial damage and pyroptosis following CME. **(A)** Transfection with pcDNA3.1-TUG1 plasmids significantly increased TUG1 level in H9C2 cells (*n* = 3, ^*^P < 0.05, ^**^P < 0.01, ^***^P < 0.001). **(B)** Transfection with AAV-pcDNA3.1-TUG1 significantly increased myocardial TUG1 level following CME (*n* = 10). **(C)** Echocardiography was used to assess cardiac function and to quantify LVEF (*n* = 10). **(D)** HBFP staining was used to measure microinfarct size (×200; scale bar=50 μm) (*n* = 5). **(E)** cTnI parameters for rats in each group (*n* = 10). **(F)** Western blotting was used to quantify myocardial NLRP3, ASC, caspase-1 p20, GSDMD, IL-1β, and IL-18 protein levels with GAPDH as a loading control (*n* = 3). Data are mean ± SEM from three or more independent experiments. ^*^P < 0.05, ^**^P < 0.01, ^***^P < 0.001.

QRT-PCR results suggest that intervention of pcDNA3.1-TUG1 plasmid and AAV-pcDNA3.1-TUG1 significantly elevated TUG1 expression *in vitro* (Figure 2A) and *in vivo* (Figure 2B). TUG1 overexpression improved LVEF following CME (Figure 2C). HBFP staining showed less micro infarct size in the CME+Ad-TUG1 group compared with the CME+Ad-NC group (Figure 2D). Serum cTnI concentration in the CME+Ad-TUG1 group was lower than in the CME+Ad-NC group (Figure 2E). Moreover, immune blotting results revealed a considerable decrease in the pyroptosis-associated proteins (NLRP3, ASC, caspase-1 p20, GSDMD, IL-1β, and IL-18) in the CME+Ad-TUG1 group compared with the CME+Ad-NC group (Figure 2F). These results indicate that TUG1 overexpression attenuates CME-induced myocardial injury and NLRP3 inflammasome-mediated pyroptosis.

### Knockdown of lncRNA TUG1 Aggravates Myocardial Injury and Pyroptosis Following CME

**Figure 3:**
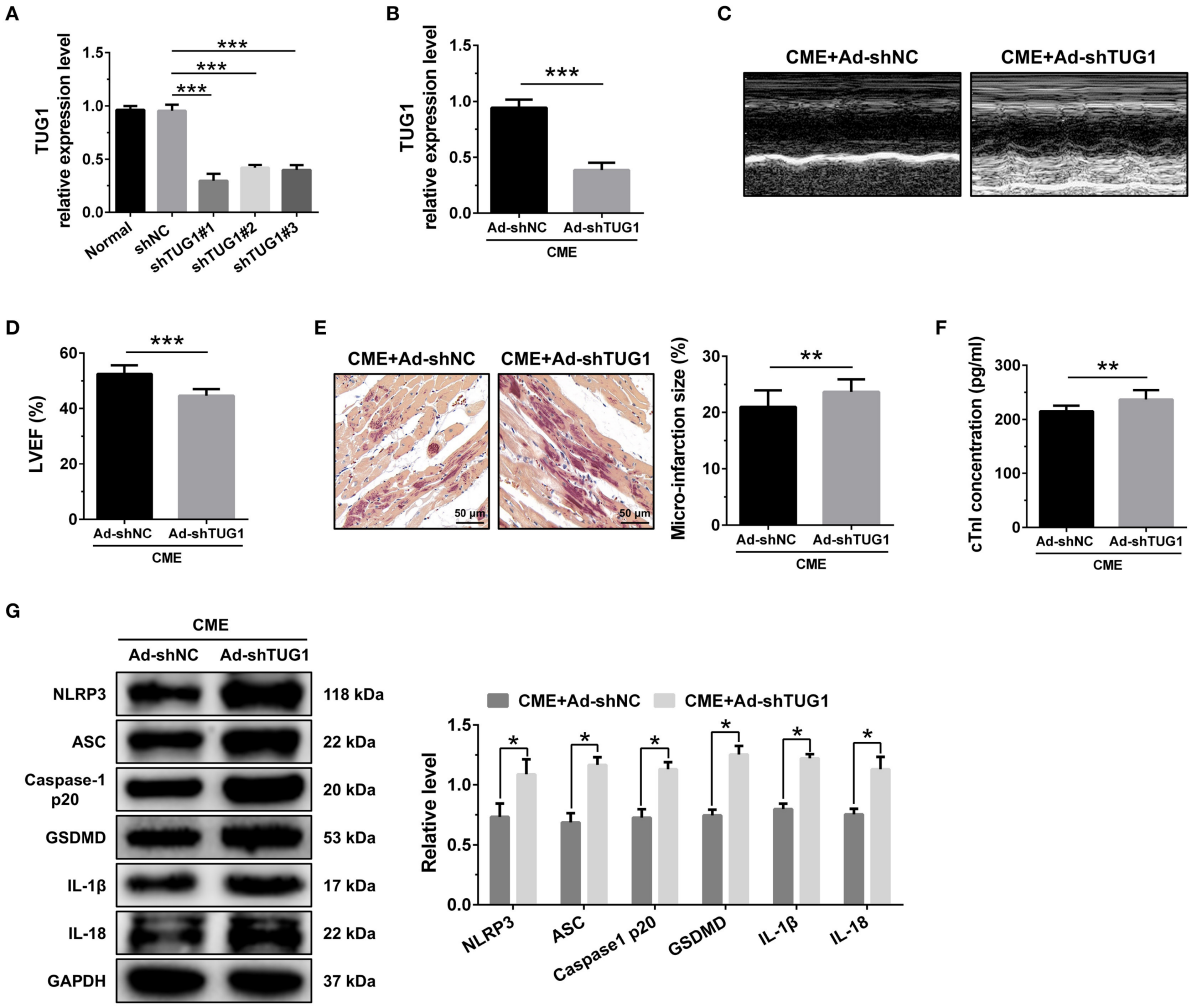
TUG1 depression exacerbates myocardial damage and pyroptosis following CME. **(A)** TUG1 shRNAs successfully decreased TUG1 level in H9C2 cells (*n* = 3). **(B)** Transfection with AAV-TUG1-shRNA significantly decreased myocardial TUG1 level following CME (*n* = 10). **(C)** Echocardiography was used to assess cardiac function and to quantify **(D)** LVEF (*n* = 10). **(E)** HBFP staining was used to measure microinfarct size (×200; scale bar = 50 μm) (*n* = 10). **(F)** cTnI parameters for rats in each group (*n* = 10). **(G)** Western blotting was used to quantify myocardial NLRP3, ASC, caspase-1 p20, GSDMD, IL-1β, and IL-18 protein levels with GAPDH as a normalization control (*n* = 3). Data are mean ± SEM from three or more independent experiments. ^*^P < 0.05, ^**^P < 0.01, ^***^P < 0.001.

The obtained results of QRT-PCR revealed that TUG1-shRNAs considerably inhibited the expression of LOX-1 mRNA in vitro, and shRNA#1 revealed the highest knockdown efficiency (Figure 3A). Transfection with AAV-shTUG1#1 significantly decreased the TUG1 level in the myocardium following CME (Figure 3B). Therefore, AAV-shTUG#1 was selected for subsequent experiments.

The knockdown of TUG1 significantly reduced LVEF following CME (Figures 3C,D). HBFP staining showed a larger microinfarct size in the CME+Ad-shTUG1 group compared with the CME+Ad-shNC group (Figure 3E). Serum CTNI concentration in the CME+Ad-shTUG1 group was higher than in the CME+Ad-shNC group (Figure 3F). Moreover, immune blotting results revealed a considerable elevation in the pyroptosis-associated proteins in the CME+Ad-shTUG1 group compared with the CME+Ad-shNC group (Figure 3G). The results demonstrate that TUG1 knockdown could aggravate CME-induced myocardial injury and NLRP3 inflammasome-mediated pyroptosis.

### MiR-186-5p Is a Direct Target of TUG1 and Regulates CME-Induced Myocardial Injury

**Figure 4:**
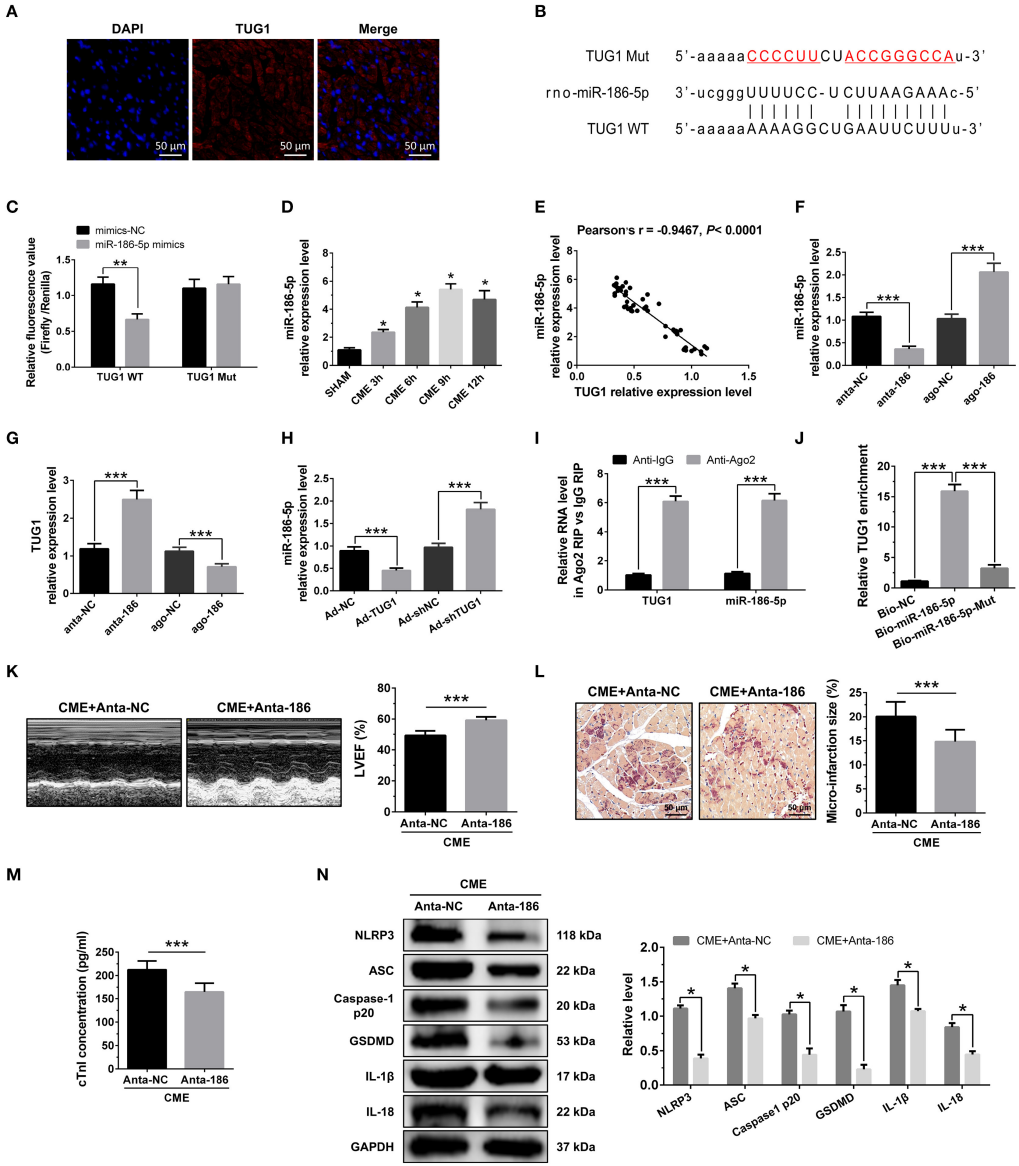
MiR-186-5p is a direct target of TUG1 and regulates CME-induced myocardial injury. **(A)** Representative images of fluorescence in situ hybridization (FISH) for TUG1 in CME heart tissue (magnification, ×400; bar = 50μm). **(B)** The predicted binding region between miR-186-5p and TUG1 was predicted by using bioinformatics analysis. **(C)** Relative luciferase activity in WT-TUG1 and MUT-TUG1. **(D)** Myocardial miR-186-5p levels were assessed via qRT-PCR (*n* = 10), ^*^P < 0.05 vs. SHAM group. **(E)** MiR-186-5p level was negatively correlated with TUG1 level in heart tissue. **(F)** MiR-186-5p was silenced or overexpressed with antagomiR or agomiR; qRT-PCR was used to detect the transfection efficiency (*n* = 3). **(G)** The expression levels of TUG1 were tested in miR-186-5p-downregulated or miR-186-5p-overexpressed rats with qRT-PCR (*n* = 10). **(H)** The expression levels of miR-186-5p were tested in TUG1-downregulated or TUG1-overexpressed rats with qRT-PCR (*n* = 10). **(I)** RIP assay was carried out to demonstrate the combination between TUG1 and miR-186-5p. **(J)** Pulldown assay was applied to prove the binding relation between TUG1 and miR-186-5p. **(K)** LVEF was assayed by echocardiography (*n* = 10). **(L)** HBFP staining was used to measure micro infarct size (×200; scale bar=50 μm) (*n* = 10). **(M)** cTnI parameters for rats in each group (*n* = 10). **(N)** Western blotting was used to quantify myocardial NLRP3, ASC, caspase-1 p20, GSDMD, IL-1β, and IL-18 protein levels with GAPDH as a normalization control (*n* = 3). Data are mean ± SEM from three or more independent experiments. ^*^P < 0.05, ^**^P < 0.01, ^***^P < 0.001.

RNA FISH results revealed that TUG1 is an lncRNA that is abundant in the cytoplasm (Figure 4A) and that lncRNAs of this kind often function through a ceRNA mechanism. Bioinformatics predictions using StarBase V2.0 (http://starbase.sysu.edu.cn/starbase2/index.php) indicate that the TUG1 sequence contained a miR-186-5p binding site. To evaluate the direct interactions between miR-186-5p and the TUG1 3'UTR, we mutated the miR-186-5p binding site in TUG1 to generate Luc-TUG1-mut (Figure 4B). Functional (miR-186-5p mimics) and invalid (mimics-NC) miR-186-5p analogs were constructed. The findings of the Luciferase reporter gene assay revealed that mutant TUG1 reversed the luciferase activity results relative to those of WT TUG1 when there was a sufficient level of functional miR-186-5p mimics, proving that TUG1 directly targets miR-186-5p (Figure 4C). On the other side, we examined the level of miR-186-5p expression *in vivo*. MiR-186-5p presented a time-dependent increase after CME (Figure 4D) and was negatively associated with the TUG1 expression (Figure 4E). The obtained results of RT-PCR proved that miR-186-5p was highly expressed via miR-186-5p agomiR but was silenced by miR-186-5p antagomiR in heart tissue (Figure 4F). Knockdown of miR-186-5p elevated the TUG1 level in the myocardium undergoing CME, and miR-186-5p overexpression suppressed TUG1 (Figure 4G). Next, the elevated TUG1 expression level potentially suppressed miR-186-5p expression in the myocardium following CME, and knockdown of TUG1 elevated miR-186-5p level (Figure 4H). The RIP assay demonstrated that both TUG1 and miR-186-5p tended to enrich in Ago2-immunoprecipitation (Figure 4I). What's more, the RNA-pull down assay demonstrated that TUG1 could only be pulled down by the WT biolabeled miR-186-5p, but not the mutated oligos (Figure 4J). It has been validated that TUG1 functions as a ceRNA when it competitively binds with miR-186-5p in CME.

To identify the miR-186-5p role in CME, we performed loss-of-function experiments. MiR-186-5p depression significantly improved LVEF following CME (Figure 4K). Knockdown miR-186-5p reduced micro infarct size significantly (Figure 4L). Knockdown miR-186-5p decreased serum cTnI concentration following CME (*P* < 0.05, Figure 4M). Moreover, immune blotting results revealed a considerable decrease in the pyroptosis-associated proteins in the CME+anta-186 group compared with those in the CME+anta-NC group (Figure 4N). These results reveal that knockdown of miR-186-5p could improve CME-triggered myocardial injury and NLRP3 inflammasome-mediated pyroptosis.

### Overexpression of TUG1 Suppressed Cardiomyocyte Pyroptosis Through Regulation of miR-186-5p

**Figure 5:**
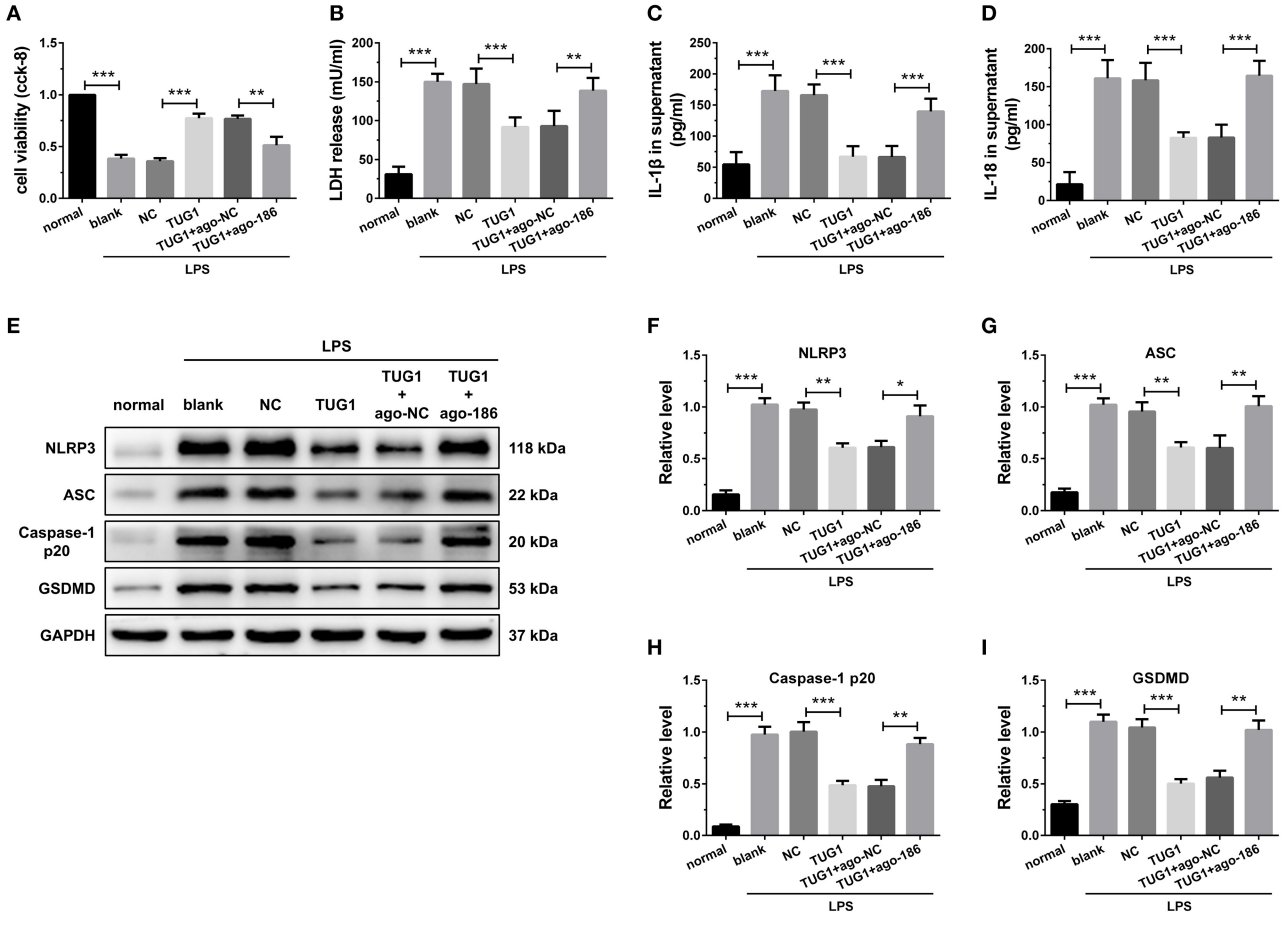
Overexpression of TUG1 suppressed cardiomyocyte pyroptosis through regulation of miR-186-5p. **(A)**) A CCK-8 assay used to gauge H9C2 cardiomyocyte viability (*n* = 3). Supernatant **(B)** LDH, **(C)** IL-1β, and **(D)** IL-18 levels were measured via ELISA (*n* = 6). **(E-I)** Western blotting was used to quantify cardiomyocyte NLRP3, ASC, caspase-1 p20, and GSDMD protein levels with GAPDH as a loading control (*n* = 3). Data are mean ± SEM from three or more independent experiments. ^*^P < 0.05, ^**^P < 0.01, ^***^P < 0.001.

To investigate the basic mechanism, LPS-induced *in vitro* inflammatory model was established in the H9C2 cardiomyocytes. The results of CCK-8 revealed that elevated expression of miR-186-5p decreased the elevated cell viability caused by TUG1 overexpression (Figure 5A). Meanwhile, the overexpression of miR-186-5p increased TUG1; overexpression reduced LDH, IL-1β, and IL-18 release (Figures 5B–D). Moreover, the results of Western blotting revealed that the expression level of pyroptosis-associated proteins was dramatically down regulated by overexpression of TUG1, which was reversed by overexpression of miR-186-5p (Figures 5E–I). These results demonstrate that overexpression of TUG1 improves LPS-induced cardiomyocyte injury and pyroptosis by sponging miR-186-5p.

### XIAP Is a Direct Target of miR-186-5p and Regulates CME-Induced Myocardial Injury

**Figure 6:**
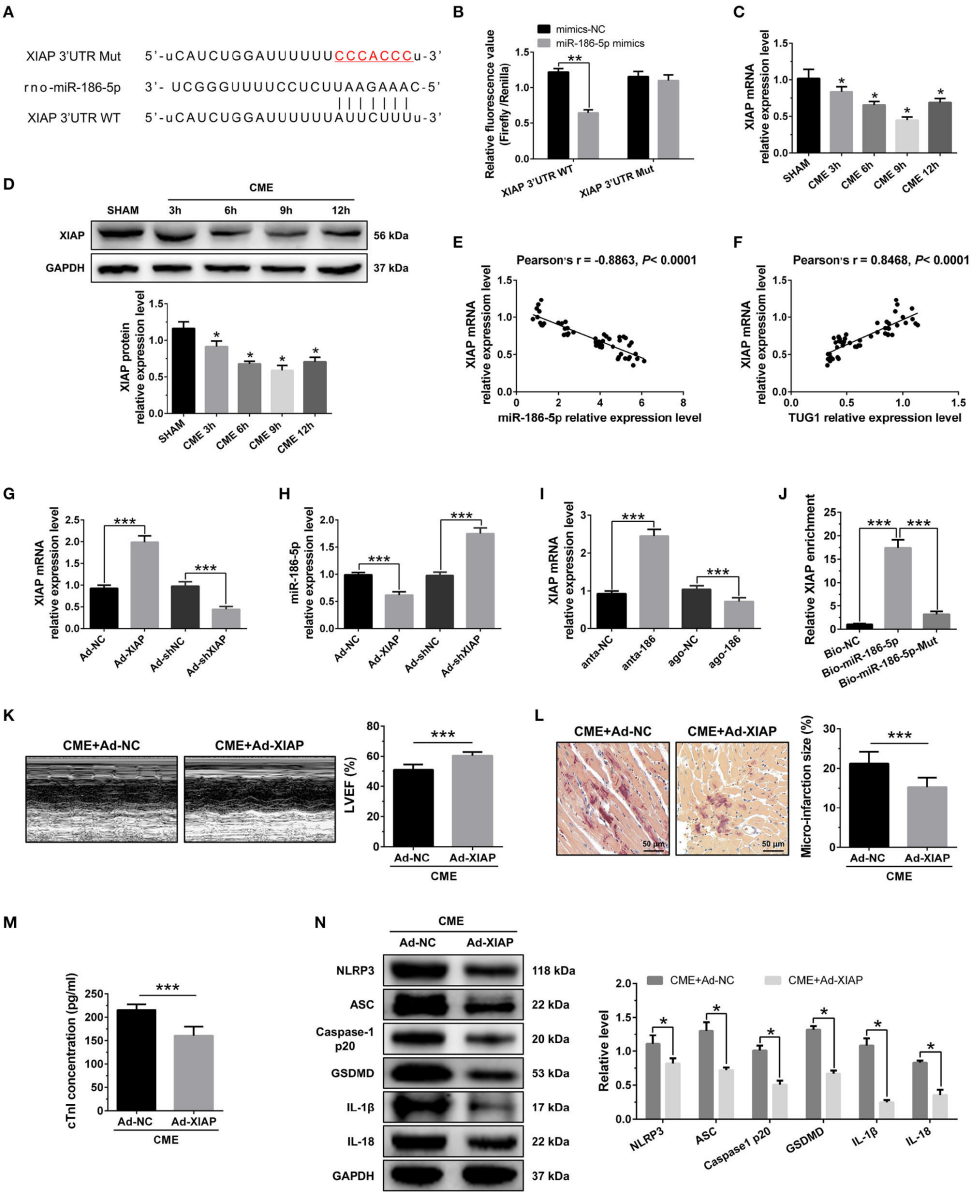
XIAP is a direct target of miR-186-5p and regulates CME-induced myocardial injury. **(A)** The binding sites between miR-186-5p and XIAP were predicted by using bioinformatics analysis. **(B)** Relative luciferase activity in WT-XIAP and MUT-XIAP. **(C)** Myocardial XIAP mRNA levels were assessed via qRT-PCR (*n* = 10), ^*^P < 0.05 vs. SHAM group. **(D)** Myocardial XIAP protein levels were assessed via Western blotting (*n* = 3), ^*^P < 0.05 vs. SHAM group. **(E)** XIAP mRNA level was negatively correlated with miR-186-5p level in heart tissue. **(F)** XIAP mRNA level was positively correlated with TUG1 level in heart tissue. **(G)** XIAP was silenced or overexpressed with AAV-shXIAP or AAV-pcDNA3.1-XIAP. qRT-PCR was used to detect the transfection efficiency (*n* = 3). **(H)** The expression levels of miR-186-5p were tested in XIAP-down regulated or XIAP-overexpressed rats with qRT-PCR (*n* = 10). **(I)** The expression levels of XIAP mRNA were tested in miR-186-5p-downregulated or miR-186-5p-overexpressed rats with qRT-PCR (*n* = 10). **(J)** Pulldown assay was conducted to prove the combination between miR-186-5p and XIAP mRNA. **(K)** Overexpression of XIAP improved cardiac function. LVEF was assayed by echocardiography (*n* = 10). **(L)** HBFP staining was used to measure microinfarct size (×200; scale bar=50 μm) (*n* = 10). **(M)** cTnI parameters for rats in each group (*n* = 10). **(N)** Western blotting was used to quantify myocardial NLRP3, ASC, caspase-1 p20, GSDMD, IL-1β, and IL-18 protein levels, with GAPDH as a normalization control (*n* = 3). Data are mean ± SEM from three or more independent experiments. ^*^P < 0.05, ^**^P < 0.01, ^***^P < 0.001.

The bioinformatics tools were employed for the prediction of miR-186-5p target genes via TargetScan (http://www.targetscan.org/vert_72/). XIAP was evaluated as a candidate miR-186-5p target (Figure 6A). As depicted in Figure 6B, the luciferase activity was considerably decreased when cell transfection was carried out with miR-186-5p mimics in WT-XIAP. However, no significant difference was found in MUT-XIAP. Additionally, miR-186-5p target gene XIAP, either at the gene or protein level, was considerably decreased in the CME group and reached the lowest expression level 9 h after CME (Figures 6C,D). There was a negative correlation between XIAP mRNA and miR-186-5p level in heart tissue (Figure 6E) although positively correlated with TUG1 level (Figure 6F). XIAP was overexpressed by AAV-pcDNA3.1-XIAP but was silenced by AAV-shXIAP in the myocardium (Figure 6G). Further experiments show that overexpression of XIAP significantly down regulated the miR-186-5p level, and inhibition of XIAP remarkably up regulated the miR-186-5p level in heart tissue (Figure 6H). Moreover, it was observed that the expression of XIAP mRNA was significantly down regulated by overexpression of miR-186-5p and was remarkably up regulated via inhibition of miR-186-5p in heart tissue (Figure 6I). The pull down assay was performed, and it revealed that XIAP could only be pulled down via WT biolabeled miR-186-5p and not the mutated oligos (Figure 6J). These results reveal that miR-186-5p directly targeted and negatively regulated XIAP.

To investigate the role of XIAP in CME, we performed gain-of-function experiments. The elevated expression of XIAP significantly improved LVEF following CME (Figure 6K). The elevated expression of XIAP decreased micro infarct size significantly (Figure 6L). Overexpression of XIAP decreased serum cTnI concentration following CME (Figure 6M). Moreover, Western blotting results showed a significant decrease in the levels of pyroptosis-associated proteins in the CME+Ad-XIAP group than the CME+Ad-NC group (Figure 6N). These results reveal that XIAP overexpression could improve CME-initiated myocardial injury and NLRP3 inflammasome-mediated pyroptosis.

### Attenuation of miR-186-5p Suppressed Cardiomyocyte Pyroptosis Through Regulation of XIAP

**Figure 7:**
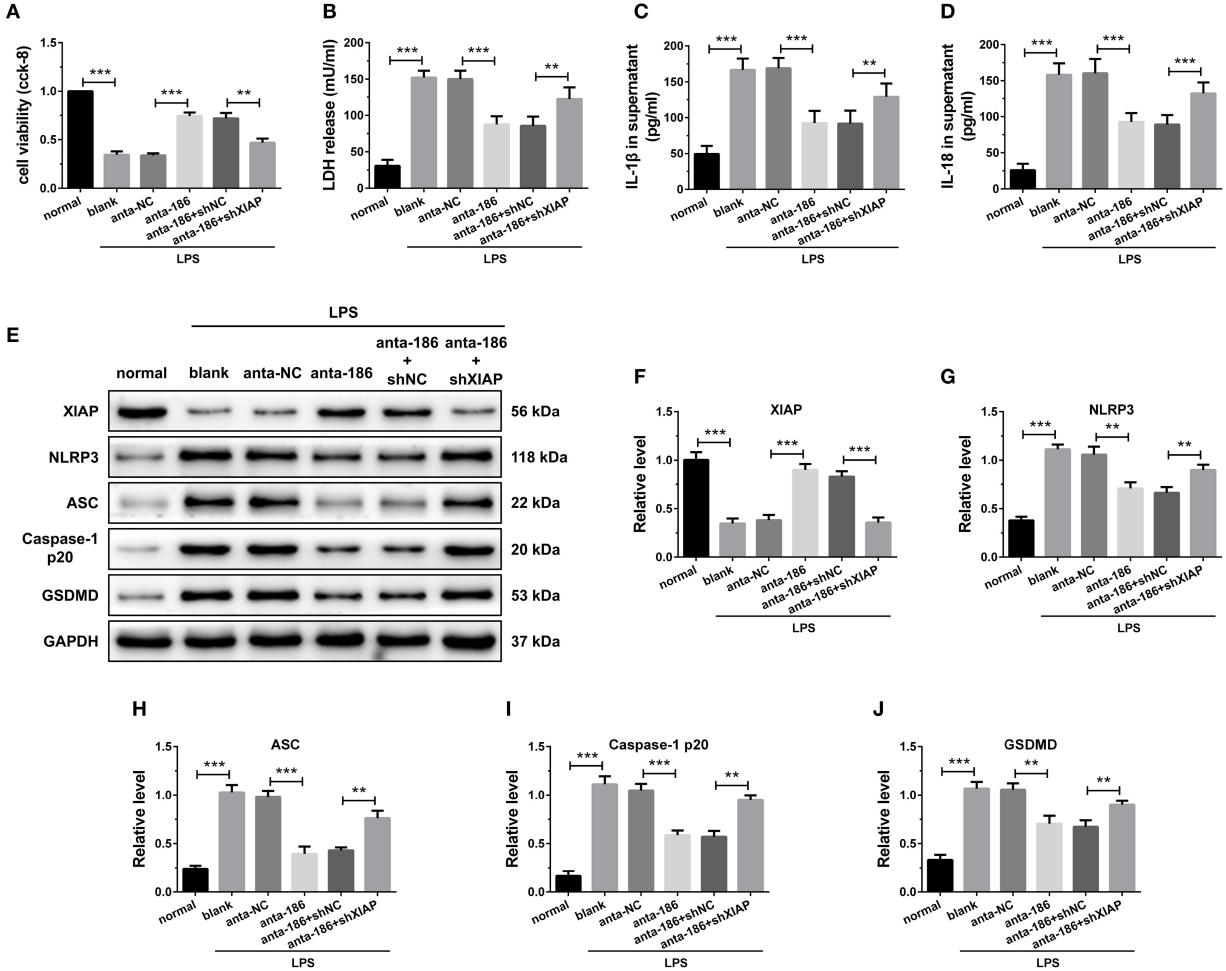
Inhibition of miR-186-5p suppressed cardiomyocyte pyroptosis through regulation of XIAP. **(A)** A CCK-8 assay used to gauge H9C2 cardiomyocyte viability (*n* = 3). Supernatant **(B)** LDH, **(C)** IL-1β, and **(D)** IL-18 levels were measured via ELISA (*n* = 6). **(E-J)** Western blotting was used to quantify cardiomyocyte XIAP, NLRP3, ASC, caspase-1 p20, and GSDMD protein levels with GAPDH as a loading control (*n* = 3). Data are mean ± SEM from three or more independent experiments. ^*^P < 0.05, ^**^P < 0.01, ^***^P < 0.001.

The effects of miR-186-5p and XIAP were then further investigated by knockdown miR-186-5p and XIAP simultaneously in H9C2 cells. When the miR-186-5p was inhibited, the cell viability was significantly up regulated and the LDH, IL-1β, and IL-18 release were remarkably reduced compared with the anta-NC control. However, the depression of XIAP dramatically reversed these effects (Figures 7A–D). The results of immune blotting revealed that knockdown of miR-186-5p increased XIAP protein level in cardiomyocytes, and shXIAP decreased XIAP protein level. Expression of pyroptosis-associated proteins was dramatically down regulated by the inhibition of miR-186-5p, which were remarkably reversed by co-transfection with shXIAP (Figures 7E–J). These results indicate that the inhibition of miR-186-5p could decrease the hypoxia-initiated cardiomyocyte injury and pyroptosis, and the process might be through the regulation of XIAP.

### Overexpression of TUG1 Suppressed Cardiomyocyte Pyroptosis Through Regulation of XIAP

**Figure 8:**
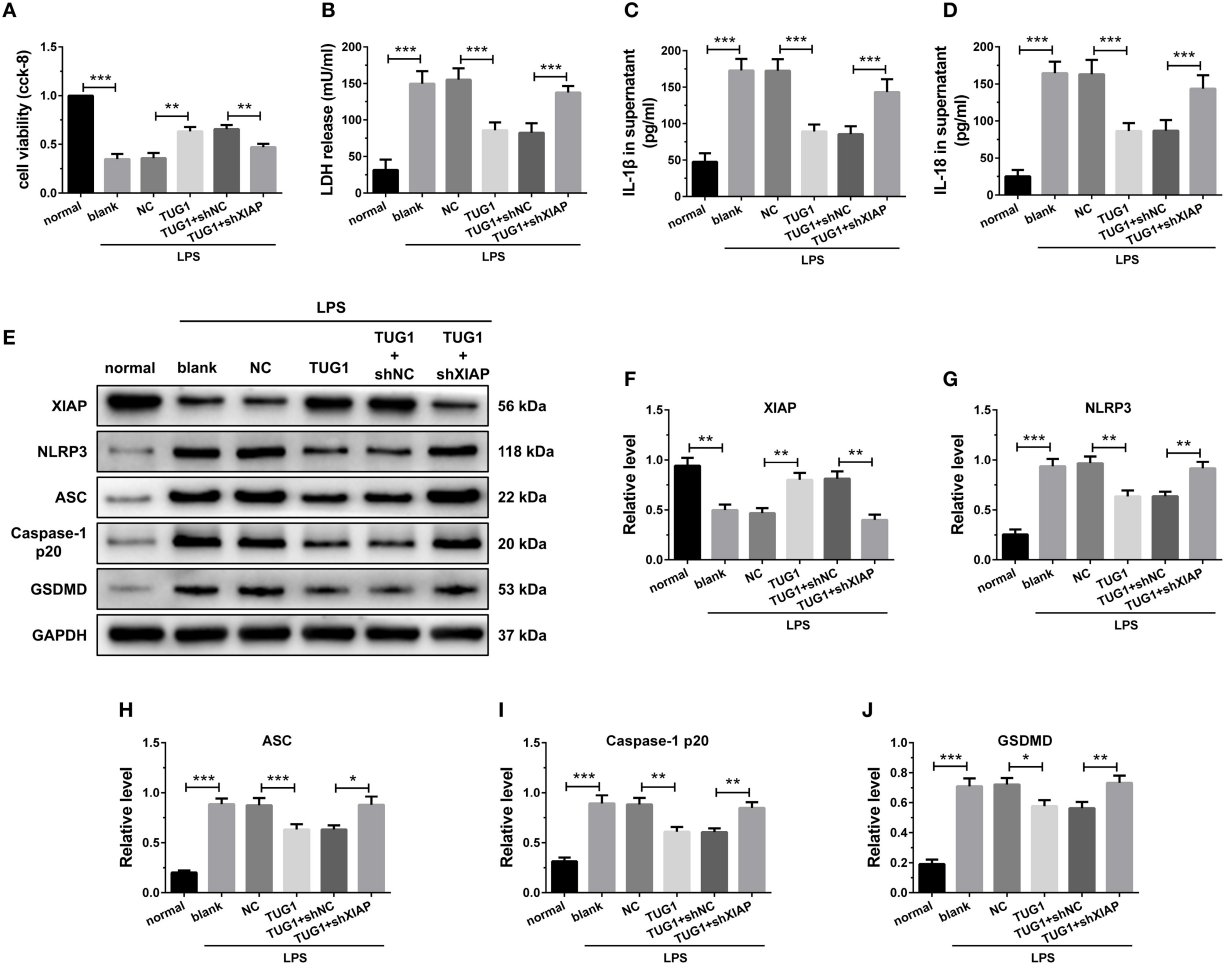
Overexpression of TUG1 suppressed cardiomyocyte pyroptosis through regulation of XIAP. **(A)** A CCK-8 assay used to gauge H9C2 cardiomyocyte viability (*n* = 3). Supernatant **(B)** LDH, **(C)** IL-1β, and **(D)** IL-18 levels were measured via ELISA (*n* = 6). **(E-J)** Western blotting was used to quantify cardiomyocyte XIAP, NLRP3, ASC, caspase-1 p20, and GSDMD protein levels with GAPDH as a loading control (*n* = 3). Data are mean ± SEM from three or more independent experiments. ^*^P < 0.05, ^**^P < 0.01, ^***^P < 0.001.

The results of CCK-8 reveal that knockdown of XIAP decreased the elevated cell viability caused by TUG1 overexpression (Figure 8A). Meanwhile, knockdown of XIAP increased TUG1 overexpression and reduced LDH, IL-1β, and IL-18 release (Figures 8B–D). The TUG1 overexpression increased the XIAP protein level in cardiomyocytes, and shXIAP decreased the XIAP protein level. Moreover, the expression level of pyroptosis-associated proteins was dramatically down regulated by overexpression of TUG1, which was reversed by depression of TUG1 (Figures 8E–J). The results demonstrate that overexpression of TUG1 suppresses LPS-induced cardiomyocyte pyroptosis by regulating XIAP.

## Discussion

CME is an iatrogenic complication of PCI (20). Clinical and preliminary research studies demonstrate that the degree of abnormal left ventricular systolic function caused by CME does not match the degree of blood-flow disorder (21). Dörge et al. (22) also reveal that cardiac function was increasingly impaired although the total infarct size of myocardial tissue is lower than 5%, which cannot elucidate the increasing dysfunctions of the heart. In addition to reduced blood flow, there must be other reasons leading to myocardial damage following CME. The existing evidence demonstrates that myocardial inflammatory response significantly contributes to CME-mediated myocardial damage. Chen et al. (23) reveal that inhibition of the NF-κB pathway alleviated myocardial inflammatory response and cardiac dysfunction after CME in rats. Other research discovered that blockade of TNF-α contributed to improving cardiac dysfunction after CME in minipigs (24). Our previous study proved that NLRP3 inflammasome-mediated cardiomyocyte pyroptosis, a form of inflammatory programmed cell death, contributes to CME-induced myocardial damage (25). In the current study, the expression level of pro-inflammatory cytokines (IL-1β and IL-18) and pyroptosis-associated proteins (NLRP3, ASC, caspase-1 p20, and GSDMD) in the myocardium were significantly elevated after CME, and these results are consistent with previous studies. Moreover, we first reported that the time point of the most severe degree of pyroptosis injury was 9 h post-CME, and then the damage gradually recovered. This phenomenon may be related to the gradual opening and formation of coronary collateral circulation to improve the damaged myocardium. The gradual activation of an anti-inflammatory effect may also be involved.

Recently, the newly emerging field of lncRNA research has attracted a great deal of interest. In the beginning, the research of lncRNA focused on the field of tumorigenesis (26). However, with the deepening of research, the regulatory role of lncRNA in cardiovascular disease has been wildly reported, e.g., a long inter genic noncoding RNA (Linc RNA), Linc1405, has been found to promote cardiac mesoderm specification and cardio genesis (27). LncRNA XIST was significantly up regulated in the heart undergoing ischemia-reperfusion (I/R), and XIST interfering relieved myocardial I/R injury (28). Also, lncRNA microarray analyses performed in myocardial infarction (MI) (29) and heart failure (HF) (30) provide an experimental basis for future investigation of lncRNAs implicated in heart diseases. However, the lncRNA role is not clearly understood in the context of CME. In the present study, lncRNA TUG1 was down regulated in heart tissue following CME. Moreover, the TUG1 expression level reached the lowest value at 9 h post-CME and was negatively correlated with the expression of NLRP3, IL-18, and IL-1β mRNA. To verify whether TUG1 participates in the progression of CME-activated myocardial damage, we performed loss- and gain-of-function experiments. The results show that TUG1 overexpression alleviates CME-induced myocardial injury and NLRP3 inflammasome-mediated cardiomyocyte pyroptosis. All the above results suggest that TUG1 contributes to the promotion of myocardial injury and pyroptosis following CME.

The mechanism of lncRNAs regulating pathophysiological processes is complex and can regulate gene expression at multiple levels. For example, lncRNA can directly act on chromatin (DNA molecule or histone) and then change their structure and ultimately affect gene expression (31). It is reported that lncRNAs can act as a “scaffold,” connecting transcription factors or coregulators and gene promoter regions to regulate gene expression (32). Besides this, in the cytoplasm, lncRNA can act as a competitive endogenous RNA (ceRNA), which can adsorb miRNAs that can bind to it, thereby affecting the regulation of miRNAs on downstream mRNAs (33). It is very difficult to predict the mechanism that controls the regulatory function of a particular lncRNA; thus, understanding lncRNA functions is a challenging task. Scientists have recently found that the subcellular localization of lncRNA is closely associated with its biological function. Previous studies show that lncRNAs located in the cytoplasm can act as a molecular sponge to adsorb miRNAs (33, 34). Many experiments prove that lncRNAs can serve as a competitive platform for miRNAs and mRNAs, which allow it to play an important role in CVDs. LncRNA CAREL regulates cardiomyocyte proliferation and heart regeneration in postnatal and adult heart after injury by acting as a ceRNA on miR-296 (35). LncRNA GAS5 has been shown to promote myocardial I/R injury via miR-335 sponge (36). Silent lncRNA AZIN2-sv has been found to improve MI injury via sponge miR-214 (37). In our study, we identified that TUG1 was mainly localized in the cytoplasm of cardiomyocytes in CME conditions. Inspired by the ceRNA mechanism mentioned above, we postulated that TUG1 may also function as a ceRNA. In this study, we find that miR-186-5p, a direct target of TUG1, is significantly up regulated in the myocardium following CME. The inhibition of miR-186-5p alleviates CME-induced myocardial damage and pyroptosis. Furthermore, the *in vitro* inflammatory model is established in H9C2 cardiomyocytes using LPS. *In vitro* rescue experiments suggest that TUG1 overexpression alleviates cardiomyocyte injury and pyroptosis via sponging miR-186-5p.

The miRNA is a small molecule noncoding RNA of about 21-25 nucleotides in length, and about one third of all human genes are regulated by these RNAs. It is well known that miRNAs specifically bind to target gene mRNA target mRNA 3′ un translated region (3′ UTR) and participate in the regulation of many important life activities (such as inflammation, apoptosis, autophagy, cell differentiation, and proliferation), thereby inhibiting protein synthesis at the post-transcriptional level. Numerous miRNAs have been reported for their involvement in CVDs. For instance, miR-223 is capable of inducing cardiac hypertrophy and heart failure (38). MiR-210 promotes post-MI cardiac repair in rodents (39). MiR-19a/19b reduces MI-induced cardiac damage and protects cardiac function (40). Furthermore, our previous studies also confirm that miR-142-3p and miR-486-5p are involved in CME-induced myocardial injury (41, 42). Interestingly, we also find that XIAP was the direct target of miR-186-5p according to the results of the dual luciferase reporter gene and RNA-pull down assay and can be negatively modulated by miR-186a-5p. Herein, XIAP overexpression partially rescued rats from CME-mediated myocardial injury and pyroptosis. On the molecular level, the down regulation of XIAP could abolish the protective effects of TUG1 overexpression and miR-186-5p knockdown on LPS-induced cardiomyocyte pyroptosis. The results reveal that TUG1/miR-186-5p/XIAP axis regulates CME-induced myocardial damage and cardiomyocyte pyroptosis.

**Figure 9:**
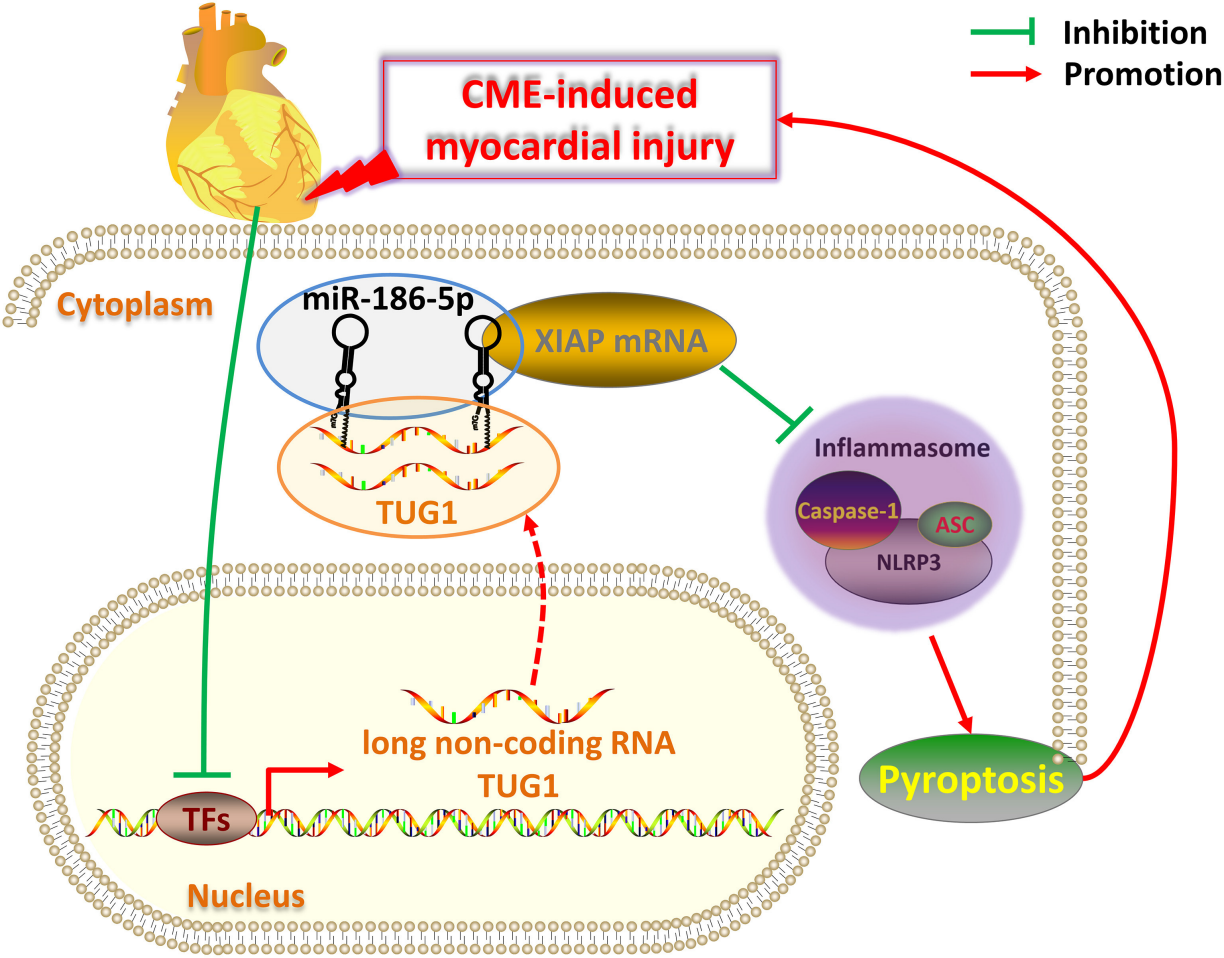
Schematic of the role of TUG1 in CME-induced myocardial injury. Once CME occurs, the expression of lncRNA TUG1 in cardiomyocytes is inhibited. The TUG1 level in the cytoplasm of cardiomyocytes significantly decreases, and the competitive adsorption of miR-186-5p weakens accordingly, which leads to the increase of miR-186-5p level. The increased miR-186-5p directly binds to the 3' UTR of XIAP mRNA and promotes its degradation, resulting in a significant decrease in XIAP expression. The decrease in XIAP leads to an increase in NLRP3 inflammasomes (composed of NLRP3, ASC, caspase-1 protein) and promotes pyroptosis, which ultimately further aggravates CME-induced myocardial injury. Breaking this TUG1/ miR-186-5p/XIAP/NLRP3 inflammasome-mediated pyroptosis pathway may be a new therapeutic target for CME-induced myocardial injury.

There are several limitations to the present study. First, the rat CME model used for this analysis is not a perfect clinical mimic of CME. It is, thus, necessary that models that more closely resemble clinical CME should be developed. Second, the mechanism of CVD regulation via lncRNAs is complex; therefore, further research related to TUG1 on CME-induced cardiac damage need to be performed. In conclusion, overexpression of lncRNA TUG1 alleviates NLRP3 inflammasome-mediated cardiomyocyte pyroptosis via sponging miR-186-5p/XIAP in CME-induced myocardial damage (Figure 9). The TUG1/miR-186-5p/XIAP axis may act as a candidate and a new therapeutic target for CME-induced myocardial injury.

## Data Availability Statement

The raw data supporting the conclusions of this article will be made available by the authors, without undue reservation.

## Ethics Statement

The animal study was reviewed and approved by the Animal Care and Use Committee of Guangxi Medical University, China.

## Author Contributions

YZ contributed to the research design and drafted the manuscript. YZ, TL, ZQ, ZC, and JH performed the experiments and analyzed data. TL and YZ contributed to proofreading and critical review of this manuscript. LL contributed to research design, oversaw all aspects of the study, and obtained funding. All authors contributed to the article and approved the submitted version.

## Conflict of Interest

The authors declare that the research was conducted in the absence of any commercial or financial relationships that could be construed as a potential conflict of interest.
